# Spatial preferences of microRNA targets in 3' untranslated regions

**DOI:** 10.1186/1471-2164-8-152

**Published:** 2007-06-07

**Authors:** William H Majoros, Uwe Ohler

**Affiliations:** 1 Institute for Genome Sciences and Policy, Departments of Biostatistics & Bioinformatics and Computer Science, Duke University, 101 Science Dr, Box 3382, Durham NC 27708, USA

## Abstract

**Background:**

MicroRNAs are an important class of regulatory RNAs which repress animal genes by preferentially interacting with complementary sequence motifs in the 3' untranslated region (UTR) of target mRNAs. Computational methods have been developed which can successfully predict which microRNA may target which mRNA on a genome-wide scale.

**Results:**

We address how predicted target sites may be affected by alternative polyadenylation events changing the 3'UTR sequence. We find that two thirds of targeted genes have alternative 3'UTRs, with 40% of predicted target sites located in alternative UTR segments. We propose three classes based on whether the target sites fall within constitutive and/or alternative UTR segments, and examine the spatial distribution of predicted targets in alternative UTRs. In particular, there is a strong preference for targets to be located in close vicinity of the stop codon and the polyadenylation sites.

**Conclusion:**

The transcript diversity seen in non-coding regions, as well as the relative location of miRNA target sites defined by it, has a potentially large impact on gene regulation by miRNAs and should be taken into account when defining, predicting or validating miRNA targets.

## Background

Recent years have seen an increased appreciation for the importance of post-transcriptional regulation in eukaryotic organisms [1,2]. The same primary transcript can lead to a number of different isoforms by processing steps such as alternative splicing [3] or polyadenylation [4]. New classes of non-coding RNA genes have been described, including the abundant and conserved class of microRNAs (miRNAs). For instance, one of the earliest identified miRNAs, let-7, is conserved across an impressively wide range of species [5], but its usage in timing of developmental transitions is employed in different species-specific settings [6]. The set of miRNAs comprises hundreds of members in mammalian organisms [7,8], and together, they are regulators of a large fraction of protein-coding genes [9-11], with an important role emerging in developmental transitions and differentiation, as well as establishing cell identity [12-14]. As important regulators of post-transcriptional gene expression, miRNAs are part of essential regulatory networks [15,16]. They have been implied as tumor suppressors [17] and oncogenes [18], and miRNA expression profiles allow for a classification between different tumors [19].

The cis-regulatory sequences of many known post-transcriptional events are located in the 3' untranslated region (3'UTR) between a stop codon and a polyadenylation site (PAS) of an mRNA. According to our current understanding, animal miRNAs follow this model and suppress protein-coding genes mostly by pairing with complementary "target sites" located in the 3'UTR, presumably leading to degradation of the transcript by cleavage or deadenylation [20], or the inhibition of translation [21], e.g. by sequestering the targeted messages into cellular compartments [22]. Current computational miRNA target site prediction algorithms [23,24] achieve a high signal-to-noise ratio (SNR) of real versus spurious hits by locating "seed matches" complementary to the 5' end of the mature miRNA, and by requiring conservation of seed matches across several species [9,10,13]. In addition, non-perfect seed matches may be complemented by more extensive base pairing of the 3' region, but this strategy leads to a smaller fraction of reliably predicted targets [11]. In rarely demonstrated cases, cleavage triggered by near-perfect complementarity [25] has been observed.

Target predictions have initially relied on ad-hoc UTR annotations (e.g., choosing 2 kb downstream of each annotated stop codon [26]), and are currently mostly based on UTRs derived from cDNA sequence repositories such as RefSeq [27]. However, alternative polyadenylation and alternative splicing can lead to changes in the 3'UTR, which opens up the possibility to specifically include or exclude individual target sites in different isoforms of a gene. The increasing number of reports addressing the high frequency of variation in the 3'UTR [28,29] and its consequences on gene regulation [30] makes it necessary to study target prediction in more detail. Here we examine how miRNA target sites are affected by alternative PAS and how they are distributed along the untranslated regions, and propose an appropriate classification scheme for target genes.

## Results

### A dataset of alternative 3' UTRs

**Figure 1:**
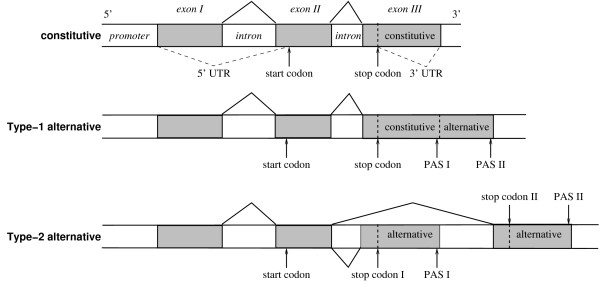
**Variants of 3' untranslated regions (UTRs)**. Shown from the top are constitutive UTRs; type-1 alternative UTRs with multiple PAS in the same exon, and type-2 alternative UTRs with multiple PAS in different exons.

We distinguish here two non-disjoint classes of alternative 3'UTRs (Figure 1): Those arising from the use of one or more PAS in the same terminal exon (type 1), and those arising from alternative terminal exons (ATE; type 2). In the first case, the UTR will consist of a 5' "constitutive" and a 3' "alternative" part. The alternative part can consist of several segments in case of multiple alternative PAS. In the case of alternative terminal exons, the different 3'UTRs will generally not share common sequence, and the choice of downstream final exons is likely coupled with suppression of the 3' splice site of the more upstream ones. As there is no constitutive shared sequence common to all isoforms, all alternative terminal exon UTRs are labeled as "alternative" parts. Tian *et al*. recently introduced the additional category of "composite" terminal exons, i.e. exons which can either be internal and spliced to a more downstream one, or terminal due to suppression of the 5' splice site and polyadenylation in the downstream intron [31]. According to our definition, such cases would fall under the larger umbrella of alternative terminal exons. We refer to the complete UTR between a stop codon and the most downstream PAS as "maximal UTR", and to the regions between alternative PAS as "segments".

We based our annotation of 3'UTRs on the databases PolyA_DB [28] and RefSeq [27]. By connecting polyA sites from polyA_DB with specific stop codons from RefSeq annotations, we arrived at a set of detailed 3'UTR annotations consistently flanked by a stop codon on the 5' end and one or more PAS on the 3' end. Our set comprised 3'UTR annotations for 11,576 human genes, including 4,728 genes (40.8%) with alternative 3'UTRs. Alternative UTR regions corresponded to 40.9% of the total sequence annotated as 3'UTR. As our annotation was restricted to RefSeq genes and UTRs with EST coverage, this number is likely to be an underestimate, especially for the case of ATEs, of which our set included only 88 cases. Anecdotal evidence for this underestimate was also provided by our UNCOVER algorithm [32] which has already detected several cases of novel ATEs in the 1% fraction of the human genome covered by the ENCODE regions [33].

### A detailed study of targets in alternative UTRs

In this study, we restricted ourselves to the most established class of miRNA targets, those with complementary seed matches. We considered one particular prediction scenario with an estimated signal-to-noise-ratio of 3.8, which was one of several alternatives examined in detail by Lewis *et al*. [10]: we required perfect Watson-Crick complementarity to bases 2–8, and conservation in alignments of 5 vertebrate species. We used the 313 human miRNAs contained in miRBase release 7 [8], which corresponded to 211 unique seeds. To predict miRNA targets in this framework, we limited our evaluation to human genes with known orthologs in the other four species. This number covers 7,161 (61.9%) of our human genes with UTR annotations. In this set of widely conserved genes, the relative fraction with alternative UTRs (3443 genes; 48.1% of the total), and as a result, the size covered by alternative UTR segments (43.4%) is somewhat larger than in the complete human set. Any biases which may be introduced by limiting evaluations to sets of genes conserved across vertebrates would however be common to many current target prediction algorithms.

**Figure 2:**
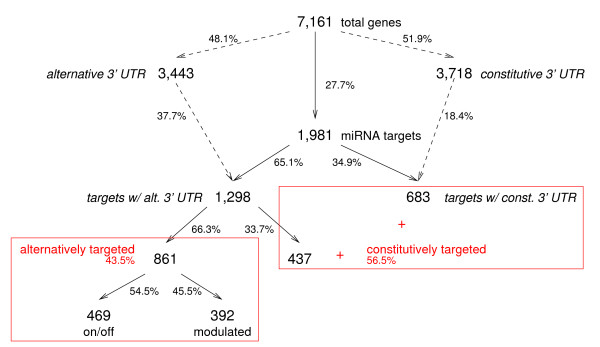
**Classification of miRNA target genes**. The total set of genes is grouped in different subclasses, defined by the presence of alternative UTRs and the location of target sites. Constitutive targets encompass predicted target genes with constitutive UTRs, as well as genes with alternative UTRs, in which all sites are located within the constitutive UTR regions. Alternatively targeted genes have at least one target site located in an alternative 3'UTR segment. On/off targets are alternative targets in which all target sites fall exclusively into alternative UTR regions; modulated targets contain alternative targets with sites in both constitutive and alternative UTR regions. Shown are the total number of genes in each class, as well as the relative size of the subclasses.

**Figure 3:**
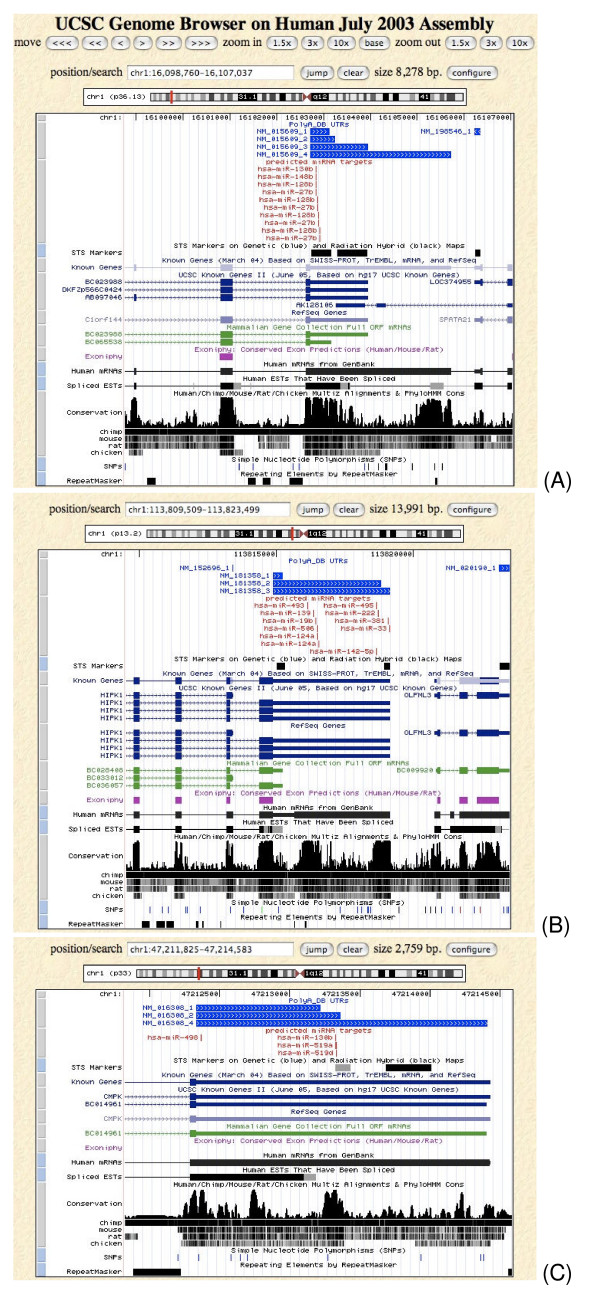
**Examples of target classes**. Screenshots from the UCSC genome browser illustrate the difference between (A) constitutive targets, (B) on/off targets and (C) alternative targets.

**Figure 4:**
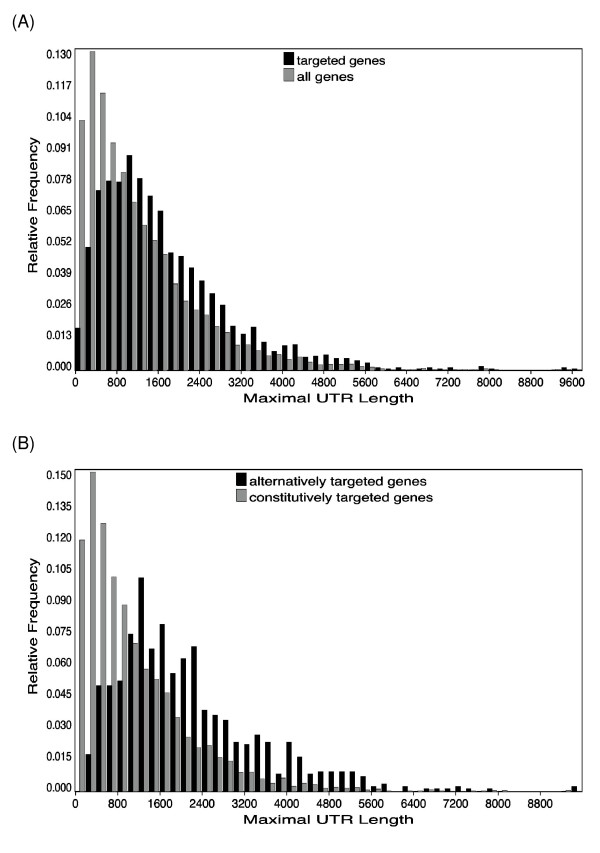
**Length distributions of maximal 3'UTRs**. (A) All genes versus miRNA targeted genes. (B) Constitutively targeted genes versus alternatively targeted genes.

We predicted 1,981 genes (27.7%) as being targeted by one or more miRNA, with an average of 3.6 target sites per targeted gene, and an average of 35.5 mRNA targets per miRNA seed. Figure 2 gives an overview of the overlap of target predictions and alternative UTRs, and Figure 3 shows examples of target sites in genes with alternative UTRs. Twice as many genes with alternative UTRs are miRNA targets when compared to genes with one PAS. Comparing the size distribution of UTRs (Figure 4), this effect is somewhat correlated with a larger maximal UTR size, and provides additional evidence that genes subject to any post-transcriptional control are more likely involved in several of these [13]. This is also reflected in the functional classification of targeted genes by Gene Ontology [34] (see Additional File 1). In summary, about two-thirds of miRNA target genes are also subject to transcript diversity altering their 3'UTRs.

Targeted genes with alternative PAS may have target sites in constitutive and/or alternative UTR regions. In our set, 40% of the sites fell in alternative segments. To provide a more detailed picture of miRNA targeting, we classified target genes into three groups: (a) *constitutive *targets, which encompass predicted target genes without alternative PAS, as well as genes with alternative PAS, but with all sites located within the constitutive UTR regions (1,120 genes = 56.6%); (b) *on/off *targets, which are genes having alternative PAS, and in which target sites fall exclusively into alternative UTR regions (469 genes = 23.7%); and (c) *modulated *targets, which contain genes with alternative PAS, and sites fall into both constitutive and alternative UTR regions (392 genes = 19.6%). Groups (b) and (c) together form the set of *alternatively *targeted genes in which one or more target sites are located in alternative 3'UTR segments. Altogether, a total of 43.3% of genes predicted as miRNA targets have at least some target sites which are likely to not always be part of the transcript.

On/off targets are targeted by fewer miRNAs than other categories: They have an average target site density of 1.4 per kb (by definition 0 in the constitutive part, and 1.7 in the alternative part), compared to 2.9/kb (4.1 constitutive/2.4 alternative) for modulated genes. This is not an artifact of shorter on/off UTRs; the maximal UTR length distributions for both on/off and modulated targets are similar to the overall distribution of alternatively targeted genes (Figure 4). In addition, the constitutive UTR segments of alternatively targeted genes cover on average only 27% of the maximal UTR, compared to 37% in all targeted genes, which is essentially the same as the 38% observed for all genes with alternative PAS. We also examined whether individual microRNAs had a preference for targeting genes in any of the three classes; however, a chi-square test at the significance level of 0.05 did not detect any such case. The Supplementary Material (Additional File 1) provides target enrichments for functional categories as given by the Gene Ontology. The total set of all targets has been previously observed to be somewhat broadly enriched for regulatory/signaling proteins [26], and some of the categories become more pronounced when analyzing the different constitutive and alternative target classes separately.

**Figure 5:**
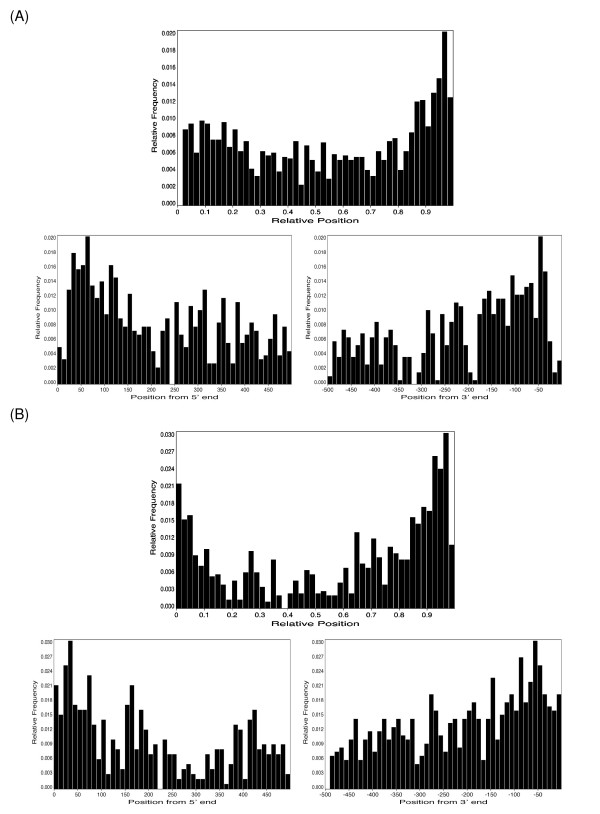
**Distribution of target site locations along 3'UTRs**. (A) Constitutive segments. In the top panel, site locations are normalized by the UTR length and given as relative position between 0 and 1. In the bottom panel, absolute site locations within the first and last 500 nt of the UTR segments are shown. Hits are binned in 10 nt intervals. (B) Alternative segments. Top and bottom panels are as described for (A).

A striking observation arose when we looked at target locations in alternative UTRs: Target locations are not evenly distributed throughout the whole UTR, but peak near the location of the PAS. This is true both for constitutive (Figure 5A) as well as alternative segments (Figure 5B) of 3'UTRs. We investigated whether this may be caused by an overlap of miRNA seed matches with polyadenylation sequence motifs; in two cases, the final pentamer of one of the canonical polyadenylation motifs accepted by polyA_DB corresponded to the beginning of a miRNA seed match (miR-205 and miR-299-5p). Looking at the distribution of all target sites for each microRNA (data not shown), this preference is due to a general bias and not caused by individual miRNAs or target mRNAs with many target sites close to the PAS. In addition to enrichment around polyA sites, the region after the stop codon is also frequently targeted; however, a close-up of the target site distribution (Figure 5A and 5B; lower panels) shows that the regions at the immediate beginning and end of UTR segments are comparatively depleted in target sites, in particular the first 20 nucleotides after the stop codon.

## Discussion

The expression of genes is fine-tuned and coordinated on a number of levels. The results in this study point to one piece in this puzzle of interactions among post-transcriptional regulatory mechanisms. In particular, it refines the notion of targets and anti-targets [35] examined in detail by several recent studies: On/off genes may switch from being targets and anti-targets depending on their UTR. Given that alternative UTRs are often expressed in a tissue-specific manner [36], this may allow to limit the downregulation of target mRNAs to specific conditions. Alternative UTRs are thus a possible mechanism to ensure a necessary flexibility of gene regulation by miRNAs. A known example of this is the *Drosophila *gene *Tropomyosin 1 *(*Tm1*), which is targeted by the muscle-specific *miR-1 *but has a muscle-specific alternative 3'UTR which excludes the miR-1 target sites [13]. Inevitably, our current classification of target genes is subject to change due to additions to the set of mammalian microRNAs, their target sites, and known 3'UTR isoforms. However, we expect that the general distinction into constitutive and regulated target classes will hold.

It is at this point not clear what the causes for target site enrichment around polyadenylation sites are. It is conceivable that the interior of long UTRs is generally prone to form secondary structure, and thus be not as accessible to the RISC complex, than at the beginning and end. The depletion of target sites within the first 20 nucleotides immediately downstream of the stop codon suggests however that the very beginning of the 3'UTR region is a suboptimal area for targeting, as it may be obstructed by the ribosome complex. These observations do however not explain the enrichment *after *internal alternative cleavage sites, which may be due to interactions of RNA processing mechanisms. We do know that this pattern is not caused by specific microRNAs or mRNAs and rather an overall more global phenomenon.

The target prediction approach we used is strongly based on conservation, and we cannot completely exclude that our observations may be caused by artifacts, e.g. in case genomic alignments should perform better on the region around polyA sites than the rest of the UTR. Indeed, conservation plots of 3'UTRs (Additional File 2) show that the overall conservation level is lower in the interior parts of the UTRs. However, in such a case we would arguably also see a comparable increase of predicted targets right after the stop codon, and this is not the case. Any arguments that an increase in targets may be an artifact of stronger conservation is circular in general – stronger conservation could both be the cause for more predictions, or caused by the presence of functional target sites. It is still open if these positional preferences also hold for target sites which do not have perfectly complementary seed matches.

Legendre et al. have also addressed the impact of alternative UTRs on microRNA targets [37]. Our work was independently carried out, using different resources and algorithms, and we arrive at highly similar rates of alternative targets, confirming the importance of transcript isoforms on miRNA targeting. In comparison to their work, we were more stringent in our alternative UTR annotation: (a) We only consider human ESTs and do not pool ESTs of different mammalian species; (b) we only consider ESTs which include polyA tails and which can therefore be unambiguously assigned to a particular polyA site. These filters limited the number of ESTs for each UTR drastically and prevented us from assigning reliable tissue information to different isoforms. After review of our manuscript, we were also made aware of concurrent work on miRNA target site prediction and preferences [38], with similar findings but no in-depth discussion on alternative UTRs.

## Conclusion

40% of currently predicted target sites reside in regions which will not always be part of a mature mRNA, and this observation has significant implications for target predictions and their validation. To verify functional miRNA:mRNA targeting, reporter constructs should make use of the UTR isoform which is actually expressed in the condition under consideration. Furthermore, a negative prediction outcome for genes with multiple PAS may for some cases only be associated with the specific conditions or cell types that were tested – the mature mRNA may simply not contain the target site due its location in an alternative segment – and may in fact be positive in other circumstances. With both a number of miRNAs as well as 3'UTRs showing strong tissue-specific preferences [39], this can become a complicated issue. In the future, this will likely be alleviated both by miRNA expression arrays [40,41] as well as genome tiling arrays which allow us to gather detailed information on the co-expression of miRNAs and their target regions.

As more biologically validated target sites become available, we will be able to tell exactly how important the relative location within the UTR is for successful targeting. In addition to other characteristics like a propensity for AU-rich regions [42], the strong positional preference is likely to be a useful new feature for target prediction algorithms. Together, these features may provide additional information allowing us to relax or eliminate the strong conservation constraints which limit current prediction algorithms to the subset of widely conserved targets.

## Methods

### Identification of 3'UTRs

We based our annotation of 3'UTRs on version 1 of the database PolyA_DB [28] (Sep 13, 2004), which is based on the hg16 assembly of the human genome. Briefly, PolyA_DB collects candidates of human PAS by stringently aligning 3'ESTs and full-length cDNAs with apparent polyA tails to the genome, while ensuring that the presence of the polyA tail cannot be explained by fortuitous complementarity to genomic sequence. These putative 3' ends of genes are evaluated for the presence of any one out of a set of canonical polyA motifs at the appropriate distance to the 3' end. To arrive at complete UTR sequences and further increase our confidence in these annotations, we required that PAS candidates could be connected to a stop codon in the same terminal exon via overlapping EST/cDNA alignments, using gene annotations based on RefSeq. In this way, we excluded cases with unclear significance of target predictions, e.g. in which PAS candidates (and consequently, miRNA target sites) were located in exons upstream of the terminal one. Our association of polyA sites with specific stop codons gave rise to a set of detailed 3'UTRs flanked by a stop codon on the 5' end and PAS on the 3' end.

### Prediction of microRNA target sites

Prediction algorithms vary in some details, e.g. the exact location of the seed match (usually, complementary to bases 2–7 or 2–8 of the miRNA), whether only Watson-Crick base pairs or also G-U base pairs are allowed, and whether the identification of conserved targets relies on pre-computed alignments or is carried out independently in each species. Accordingly, the number of predicted targets and the associated SNR will change somewhat. miRNAs are commonly grouped into families, the members of which share the same seed and are thus predicted to target the same genes by algorithms relying on seed complementarity only. We considered one particular prediction scenario of the TargetScanS algorithm with an estimated signal-to-noise-ratio of 3.8 [10]: we required perfect Watson-Crick complementarity to bases 2–8, and conservation in alignments of 5 vertebrate species (human, mouse, rat, dog and chicken). According to a recent evaluation [24], algorithms relying on conserved seed matches, such as the TargetScanS strategy followed here, were able to correctly identify about 2/3 of known conserved target sites, i.e. in our case, the targets conserved among mammals and birds. We used the 313 human miRNAs contained in miRBase release 7 [8], which collapse into 211 families with unique seeds. We then used the UCSC genome browser 8-way MULTIZ alignments on the hg17 assembly of the human genome [43] to extract the orthologous UTRs, and scanned the alignments for perfectly conserved segments complementary to the miRNA seeds. Coordinates between the UTR set (hg16) and the alignments (hg17) were mapped with the UCSC LiftOver tool.

At the time of the initial TargetScanS algorithm, only 62 miRNA families were known; restricting our predictions to the same set of families, we can assess how our analysis compares to this smaller earlier set. (More recent publicly available sets of TargetScan predictions are not based on the same group of five conserved species and thus not amenable for comparison.) Lewis *et al*. described 5-way conserved predictions in 1,509 genes, we in 1,593, with an overlap of 852 genes (56%). It is easy to conceive that we made additional predictions due to the increased volume of available cDNA and EST sequences. In addition, of the 657 genes predicted by TargetScanS alone but not present in our analysis, 531 (81%) are not in our database of reliable complete 3'UTR annotations and are thus not predicted by default. Therefore, the predictions are in good agreement, but the discrepancies hint at the strong influence that reliable and complete UTR definitions have on the predictions of miRNA targets.

### Availability

A complete list of predictions is provided in Additional Files 3 and 4 and is also accessible on the web [44]. This website also contains the predictions in appropriate format for display in the UCSC genome browser.

## Authors' contributions

WHM implemented the UTR and microRNA target database. Both WHM and UO analyzed the data. UO conceived the study and wrote the manuscript.

## Supplementary Material

Additional file 1 Tables with enriched Gene Ontology categories of miRNA targeted genes. Click here for file

Additional file 2 Conservation plots of mammalian 3'UTRs. Click here for file

Additional file 3 List of targeted genes and coordinates of target sites, sorted by targeted gene. Click here for file

Additional file 4 List of targeted genes and coordinates of target sites, sorted by miRNA. Click here for file
