# Supplementary material for: Spatial preferences of microRNA targets in 3' untranslated regions
Source: BMC Genomics. 2007 Jun 7;8:152. doi: 10.1186/1471-2164-8-152 (PMC1904200; doi:10.1186/1471-2164-8-152)
Supplement: Additional file 1 — Tables with enriched Gene Ontology categories of miRNA targeted genes. [file 1471-2164-8-152-S1.pdf]

Supplementary Table 1: Biological process (first section), molecular function (second section), cellular component (third section) from the Gene Ontology. Significance was set at the 0.05 level; an asterisk indicates significance after applying Bonferroni correction for multiple comparisons. Observed counts / expected counts are shown at the bottom of each cell. Blank cells indicate non-significance even before Bonferroni correction.

**Targeted genes**	**Diff. targ. genes**	**Non-diff. targ. genes**	**On/off genes**	**Modul-ating genes**	**Name**
$^{*}7\times10^{-6}$ 520/446	2×10^{-3} 232/195	9×10^{-3} 288/254	4×10^{-2} 122/105	2×10^{-2} 110/90	Regulation of cellular process
$^{*}7\times10^{-5}$ 63/42		3×10^{-3} 48/33		2×10^{-2} 15/8	Cell differentiation
$^{*}9\times10^{-5}$ 136/104	6×10^{-3} 62/45	2×10^{-2} 74/59	2×10^{-2} 35/24		Negative regulation of biological process
$^{*}3\times10^{-4}$ 387/336		*3×10^{-4} 258/213			Cell communication
$^{*}4\times10^{-4}$ 482/427	2×10^{-2} 213/187	3×10^{-2} 269/243		2×10^{-2} 104/86	Regulation of physiological process
$^{*}4\times10^{-4}$ 55/37		2×10^{-3} 51/35			System development
5×10^{-3} 68/52					Vesicle-mediated transport
6×10^{-3} 14/8					Cell migration
8×10^{-3} 48/35	3×10^{-2} 23/16				Organ development
2×10^{-2} 9/5					Cell growth
	3×10^{-3} 15/7				Locomotion
		*1×10^{-3} 14/6			Embryonic development
		5×10^{-2} 60/49			Positive regulation of biological process
$^{*}4\times10^{-5}$ 150/115		*8×10^{-5} 94/65		4×10^{-2} 32/23	Transcription factor activity
$^{*}1\times10^{-4}$ 628/561	6×10^{-3} 276/242	1×10^{-2} 352/319	6×10^{-3} 154/128		Protein binding
$^{*}4\times10^{-4}$ 63/44		7×10^{-3} 37/25			Transcription cofactor activity
2×10^{-3} 17/9					Kinase regulator activity
4×10^{-3} 51/37		4×10^{-3} 33/21			Transcriptional activator activity
6×10^{-3} 35/24	5×10^{-2} 16/10				Transcriptional repressor activity
9×10^{-3} 10/5					Small protein conjugating enzyme activity
2×10^{-2}	3×10^{-2}				RNA polymerase II transcription factor activity

46/35	23/15				
4×10^{-2} 25/18		4×10^{-2} 16/10			Receptor signaling protein activity
	7×10^{-3} 11/5				Pattern binding
	2×10^{-2} 31/22				Ligase activity
	2×10^{-2} 135/115				Nucleotide binding
			3×10^{-2} 10/5		Carbohydrate binding
			4×10^{-2} 12/7		Enzyme activator activity
3×10^{-2} 24/17					Transcription factor complex
		2×10^{-2} 312/283			Membrane

Supplementary Table 2: GoMiner Gene Ontology false discovery rate results for biological process (first section), molecular function (second section), cellular component (third section). The maximum FDR was set at the 0.10 level; blank cells did not meet this significance. Entirely blank rows are those categories detected by the hypergeomteric test (Supplementary Table 1) but which were below the FDR threshold; categories in italic are those not deemed significant by the hypergeometric test.

**Targeted genes**	**Diff. targ. genes**	**Non-diff. targ. genes**	**On/off genes**	**Modul-ating genes**	**Name**
0.0000	0.0000	0.0000		0.0000	Regulation of cellular process
0.0000		0.0455			Cell differentiation
0.0000	0.0235				Negative regulation of biological process
0.0000	0.0683	0.0000			Cell communication
0.0000	0.0000	0.0483		0.0333	Regulation of physiological process
0.0000		0.0125			System development
					Vesicle-mediated transport
					Cell migration
0.0873					Organ development
0.0444					Cell growth
	0.0467		0.0900		Locomotion
0.0213		0.0579			Embryonic development
					Positive regulation of biological process
0.0714		0.0595			*Segmentation*
0.0860					*Cell adhesion*
0.0872					*Morphogenesis*
0.0881		0.0605			*Pattern specification*
	0.0560				*Viral infectious cycle*
0.0000	0.0000	0.0000			Transcription factor activity
0.0000		0.0385		0.0444	Protein binding
0.0085		0.0585			Transcription cofactor activity
0.0098					Kinase regulator activity
0.0341		0.0939			Transcriptional activator activity
0.0074					Transcriptional repressor activity
0.0313	0.0364				Small protein conjugating enzyme activity
0.0459					RNA polymerase II transcription factor activity
0.0357		0.1000			Receptor signaling protein activity
			0.0800		Pattern binding
					Ligase activity
0.0485	0.0955				Nucleotide binding
					Carbohydrate binding
					Enzyme activator activity
0.1000					*Phosphatase regulator activity*
					Transcription factor complex
					Membrane
0.0329					*Eukaryotic translation initiation factor 4F complex*
0.0714					*cAMP-dependent protein kinase complex*
