# Supplementary material for: Spatial preferences of microRNA targets in 3' untranslated regions
Source: BMC Genomics. 2007 Jun 7;8:152. doi: 10.1186/1471-2164-8-152 (PMC1904200; doi:10.1186/1471-2164-8-152)
Supplement: Additional file 2 — Conservation plots of mammalian 3'UTRs. [file 1471-2164-8-152-S2.pdf]

Additional File 2: Background conservation levels in 3' UTRs.

The graphs depict the overall 5-way sequence conservation (human/mouse/rat/dog/chicken) in UTRs. Local conservation values were calculated by sliding a 21 bp window along each UTR segment, assigning to each central position in the window the percentage of the columns within the window having perfect conservation across all 5 species. Requiring perfect conservation is motivated by the target prediction pipeline which only finds target sites completely conserved across species. Because segments are of different lengths, segments were normalized to a standard length, so that the position along the segment (x-axis) represents a relative distance from the beginning of the segment (cf. Figure 4A). The plots show the average local conservation across one of four sets of sequences: alternative targeted segments, alternative nontargeted segments, constitutive targeted segments, and constitutive nontargeted segments..

(i) Conservation computed on the segment level, i.e. averaging over all segments which are constitutive respectively alternative, separated by targeted versus non-targeted segments.

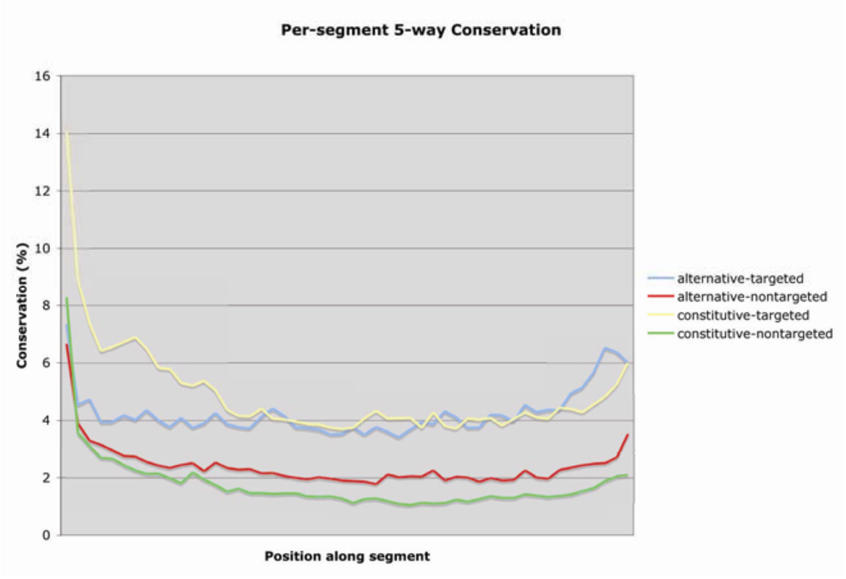

(ii) Conservation computed on gene level, i.e. averaging over all segments which are constitutive respectively alternative, separated by targeted versus non-targeted genes

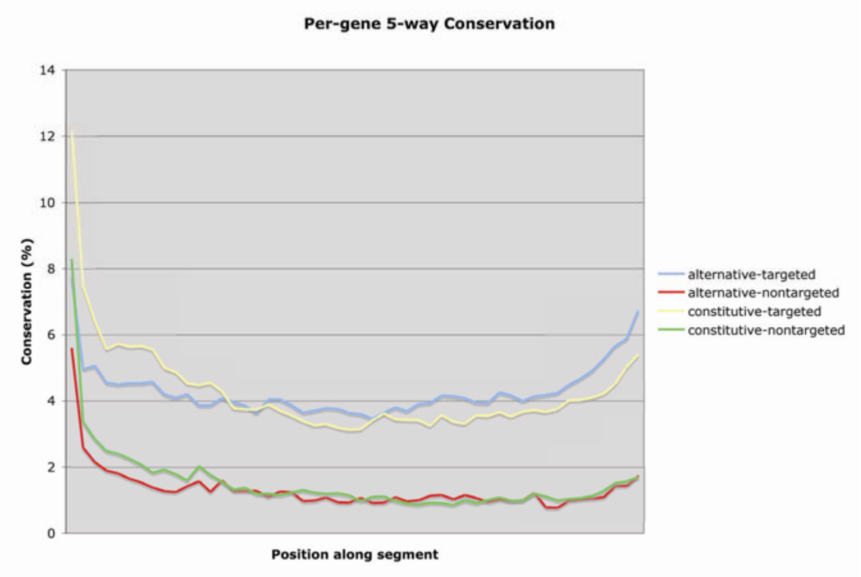

As can be seen, targeted and non-targeted genes exhibit different conservation levels, and a higher conservation is observed in the 5' area of constitutive segments (i.e. after the stop codon) than in the 5' area of alternative segments (i.e. after internal polyadenylation sites). Interestingly, the conservation level differences become more pronounced if segments are grouped on the presence of miRNA targets *anywhere* in the UTR (Panel ii) and not just on the presence of the target site in a particular segment (Panel i).
